# A precedented nuclear genetic code with all three termination codons reassigned as sense codons in the syndinean *Amoebophrya* sp. ex *Karlodinium veneficum*

**DOI:** 10.1371/journal.pone.0212912

**Published:** 2019-02-28

**Authors:** Tsvetan R. Bachvaroff

**Affiliations:** Institute of Marine and Environmental Technology, University of Maryland Center for Environmental Science, Baltimore, Maryland, United States of America; University of Cambridge, UNITED KINGDOM

## Abstract

*Amoebophrya* is part of an enigmatic, diverse, and ubiquitous marine alveolate lineage known almost entirely from anonymous environmental sequencing. Two cultured *Amoebophrya* strains grown on core dinoflagellate hosts were used for transcriptome sequencing. BLASTx using different genetic codes suggests that *Amoebophyra* sp. ex *Karlodinium veneficum* uses the three typical stop codons (UAA, UAG, and UGA) to encode amino acids. When UAA and UAG are translated as glutamine about half of the alignments have better BLASTx scores, and when UGA is translated as tryptophan one fifth have better scores. However, the sole stop codon appears to be UGA based on conserved genes, suggesting contingent translation of UGA. Neither host sequences, nor sequences from the second strain, *Amoebophrya* sp. ex *Akashiwo sanguinea* had similar results in BLASTx searches. A genome survey of *Amoebophyra* sp. ex *K*. *veneficum* showed no evidence for transcript editing aside from mitochondrial transcripts. The dynein heavy chain (DHC) gene family was surveyed and of 14 transcripts only two did not use UAA, UAG, or UGA in a coding context. Overall the transcriptome displayed strong bias for A or U in third codon positions, while the tRNA genome survey showed bias against codons ending in U, particularly for amino acids with two codons ending in either C or U. Together these clues suggest contingent translation mechanisms in *Amoebophyra* sp. ex *K*. *veneficum* and a phylogenetically distinct instance of genetic code modification.

## Introduction

The genus *Amoebophrya* is part of a diverse, ubiquitous marine alveolate lineage, based on sequences from anonymous environmental clone libraries [1–4], yet the group contains few validly described species [5]. Transcriptome surveys from two distinct *Amoebophrya* strains cultured on the core dinoflagellate hosts, *Karlodinium veneficum* and *Akashiwo sanguinea* were previously used for phylogenetic analysis [6–8]. These two *Amoebophrya* strains can be distinguished based on rDNA, protein sequences, and host specificity, but have not been formally described as different species [7,9]. In culture, *Amoebophrya* grown on core dinoflagellates takes ~ 60 hours to complete an infection cycle. The cycle starts when the dinospore enters the host cell, grows over the next 24–36 hours, and begins nuclear division [10]. Each nucleus is associated with a pair of flagella and the multinucleate *Amoebophrya* forms a compressed beehive shape inside the host. Just before emergence, the flagella begin beating, and the parasite emerges from the host by unfolding into an elongate vermiform. This transformation destroys the host. The vermiform swims away from the host remnants and, after further maturation, breaks into single celled biflagellate dinospores. A previous small scale EST survey of *Amoebophrya* sp. ex *K*. *veneficum* demonstrated AT bias for parasite transcripts, a pattern verified with PCR on naive and infected host cultures [11]. Here the codon mapping to amino acids in this parasite is further explored.

The central paradigm of molecular biology traces information from DNA to RNA to protein. *In silico* translation maps the three base codons on the mRNA to the amino acid in the protein based on a genetic code meant to mimic ribosomal translation. Each codon is translated to one amino acid, but each amino acid has between one and six possible codons. For termination, the lambda phage amber (UAG), ochre (UAA), and opal (UGA) mutants were associated with specific stop codons that could be read through due to compensatory mutations in the host [12]. More generally, contingent translation of the UGA codon as the rare amino acid selenocysteine requires information typically found in the 3’ untranslated region for accurate translation [13–15].

Aside from special cases of conditional translation or stop codon readthrough, there are also exceptions to the ‘standard’ genetic code that differently map one or more of the 64 possible codons to a single amino acid [12,16] Often these changes are observed in typical stop codons. Multiple eukaryotic lineages, including some ciliates and green algae translate stop codons UAA and UAG as glutamine [17–20] or other amino acids [21]. After this recoding, the only remaining stop codon is UGA. However, another widespread alternative genetic code, often found in mitochondria, interprets UGA as tryptophan for transcripts encoded, transcribed, and translated in the mitochondrial compartment [22,23]. In several ciliates all three typical stop codons are translated, and the stop signal appears to combine very short 3’ UTR with a conditionally translated stop codon [24,25]. Similarly in a specific trypanosomatid lineage, all three typical stop codons appear to encode amino acids [26]. The current work infers the genetic code used by *Amoebophrya* sp. ex *K*. *veneficum*, based on pairwise alignment with BLASTx and similar tools. The dinoflagellate host *K*. *veneficum* and another strain, *Amoebophrya* sp. ex *A*. *sanguinea* provide a form of control.

## Materials and methods

### Culturing, sequencing and assembly

The host-parasite cultures were grown as previously described [10]. The RNA-seq data from *Amoebophrya* sp. ex *K*. *veneficum* has been previously described and are available in the Short Read Archive at NCBI (SRX730947-8) [7]. In addition, DNA was isolated from *Amoebophrya* sp. ex *K*. *veneficum* dinospores using the CTAB method [11] and 30 million 100 base paired end reads were sequenced (Macrogen, Republic of Korea) (SRX730949). For the parasite from *Akashiwo sanguinea* only mRNA sequencing was performed (SRX730945-6). The RNA sequences were assembled using Trinity with default parameters without separation of host and parasite reads [27]. The genomic sequences were assembled using ABySS [28] with a k-mer size of 48, based on manually iterated k-mer sizes from 24 to 64 in 4 base steps to optimize the N50. SPAdes assembler [29] was also used with k-mer sizes of 21, 33, and 55. Transcripts were inferred from the SPAdes genome assembly using the StringTie pipeline based on HISAT2 read mapping [30,31].

For annotation and determination of likely reading frames, the BLASTx program was used on the *de novo* assembled transcript sequences over 1 kb long against the reference sequence protein database (ref_seq) with an evalue cut-off of less than1•10^−10^. Diamond, a rapid sequence comparison tool similar to but less sensitive than BLAST was used with the StringTie inferred transcripts as queries against the non redundant database with the–sensitive setting and an e-value cut-off of 1•10^−9^. Previously AT:GC bias was used to divide putative host and parasite sequences, with the parasite sequences having higher AT content [11]. High identity BLASTn matches against a naïve (uninfected) host EST, mitochondrial, and chloroplast data were used to identify host sequences [32–34].

### Testing alternative genetic codes

The output from BLASTx searches with different genetic codes was used in three steps to infer changes in the genetic code. The first step is to count the number of stops in the translated query sequence using the standard code and compare those results to an alternative code. The second more stringent method is to count the number of instances where these stop codons contribute to increased pairwise alignment scores with alternative genetic codes. The third is to match the amino acid aligned to the stop codon in the top BLASTx subject sequence. To determine the number and extent of sequences with typical stop codons, the BLASTx (and Diamond) alignment scores were compared with three genetic codes. Additional analyses were performed using the *Perkinsus marinus* protein coding sequences as a database and all the currently available genetic codes in BLASTx (NCBI translation tables 1–6, 9–16, 21–25). BLASTx has not implemented every described genetic code. PERL scripts were used to compare and tabulate results. For example, at positions where query UGA, UAA, UAG codons were found in the BLASTx alignment, the corresponding subject amino acid in the best alignment was extracted and tabulated using PERL. The assembled sequence data, BLAST results, and other data tables were housed in a FileMaker Pro relational database (Apple, Cupertino, CA). Manual annotation of open reading frames and basic sequence manipulation used the Sequencher 5.1 program (GeneCodes, Anne Arbor, MI). The FACIL program was used to independently identify the optimal genetic code [35].

For comparisons of UGA codons that were likely stop codons and UGA codons that were likely translated as tryptophan, two sets of sequences were collected. For likely stop codons, a set of sequences that met the following five criteria was used: 1) e-value to reference sequence database <1•10^−100^; 2) AT content ≥58%; 3) no increase in BLASTx scores with alternative genetic codes; 4) the longest sequence in the graph component for *de novo* assembled sequences; 5) no BLASTn hit to host chloroplast sequence data. A PERL script was used to select the first in frame UGA codon for these sequences and extract flanking sequence. For UGA that were likely translated as tryptophan, the UGA codons within open reading frames with increased BLASTx scores using NCBI genetic code 4 to the reference sequence database were extracted using PERL and the reading frame determined from the BLASTx result. The nucleotide context for the 5 bases before and 12 bases after UGA codons were collected and compared using two sample logo using the t-test and a p-value of 0.05 [36]. For reference, nucleotide bias for each codon position within the same transcript was simultaneously calculated. The codonw program was used for codon usage analysis [37] after the coding sequence was extracted using longorfs [38]. The tRNA from the genome survey were extracted using tRNA-scan SE [39]. Bacterial contaminant and chloroplast tRNA were subtracted based on high BLASTn identities and genomic coverage.

Inconsistent translation of UGA as a stop codon makes translation of the putative parasite sequences from *Amoebophrya* sp. ex *K*. *veneficum* difficult. To reduce bias during open reading frame searches that would impact annotation of alternatively coded transcripts, the Gene Ontology annotation used a relational process. The *Amoebophrya* sp. ex *K*. *veneficum* sequences with BLASTx alignments to NCBI reference sequences were matched with translated protein sequences from *Amoebophrya* sp. ex *A*. *sanguinea* using BLASTx. The *Amoebophrya* sp. ex *Akashiwo sanguinea* translated sequences were in turn used as queries in blast2GO [40]. The resulting GO terms were then mapped to ‘molecular process’, and compared using the Fisher test for enriched terms as implemented in blast2GO. The test was conducted in both directions–using the GO terms associated with the standard code and terms associated with alternative codes as both references and experiments. The Dynein Heavy Chain (DHC) gene family codon usage was calculated using tBLASTx with different genetic codes to infer the translated sequence then a PERL script was used to extract codons based on the amino acid alignments and the relative synonymous codon usage calculated directly from these values.

## Results

### Transcriptome of *Amoebophrya* sp. ex *Karlodinium veneficum*

After *de novo* assembly using Trinity, the RNA-seq data from *Amoebophrya* ex *K*. *veneficum* contained 228,474 sequences, of which 65,096 were longer than 1 kb and 114,938 were longer than 500 bases. Trinity reported 30,970 sequence variants within the same graph component. Based on the distribution of the AT bias histogram (S1 Fig), the sequences were divided in an approximate ratio of one third parasite and two thirds host. There were 74,549 sequences with ≥58% AT content putatively attributed to the parasite [11] of which 38,063 were longer than 500 bases, and 23,160 were longer than 1 kb. Of these sequences 12,793 had additional sequence variants reported by Trinity. Of sequences ≥58% AT content, 1,573 contained CTCAAG within the first 30 bases which matches the 3’ end of the dinoflagellate spliced leader sequence [41]. The remaining sequences with <58% AT content, or the putative host set, had 76,875 sequences longer than 500 bases, and 41,936 sequences longer than 1kb. Of these 19,170 sequences had additional sequence variants. Of all sequences <58% AT content, 11,495 contained a six base or longer spliced leader match within the first 30 bases as described above. All parasite sequences were submitted to TSA with the submission number GGWB00000000 and host sequences are under the accession GGWG00000000. In BLASTx searches against the NCBI reference sequence database using putative host sequences (<58% AT content) over 1 kb, 20,725 had hits with an e-value cutoff of 1e-10 or less. The ≥58% AT content sequences over 1 kb long produced 4,908 hits, of which 3,652 were unique after removing other sequences within the same graph component. For *Amoebophrya* sp. ex. *Akashiwo sanguinea* the RNA-seq data assembled using Trinity contained 237,751 sequences. The sequences for host and parasite could not be resolved using AT bias alone (S1 Fig). In addition, a robust naïve host library for *A*. *sanguinea* is not available for comparison.

### Features of *Amoebophrya* sp. ex *K*. *veneficum* genome assembly

The N50 size of the ABySS assembly of *Amoebophrya* sp. ex *K*. *veneficum* was 21 kb with 15,484 sequences over 1 kb long, while the SPAdes assembly had an N50 of 33 kb with 13,850 sequences over 1kb. The SPAdes coverage was distributed around 14 fold, consistent with a genome size of ~130 Mb. The ABySS and SPAdes assemblies contained a total of 123 Mb and 138 Mb in contigs over 1 kb respectively. A bacterial contaminant with a 97% SSU rDNA identity to *Kordia algicida* OT-1 was also found in the assembled data [42]. The bacterial contaminant was assembled into 80 ABySS contigs and 93 SPAdes scaffolds in the genome assemblies and spanned ~4.5 Mb of sequence. This bacterial contaminant was not well represented in the transcriptome data with few BLASTn matches to the genomic data for this bacterium. An unknown quantity of host sequence was contained within the assembled genomic data. Host chloroplast sequences were not robustly assembled by ABySS with only 16 sequences (3 over 1 kb) covering 6,613 bases of the 142 kb chloroplast data determined by Gabrielsen et al. [33] using a BLASTn identity cut-off of 90%. The SPAdes scaffolds contained over 55 kb of chloroplast data in 73 scaffolds with only 13 over 1 kb. The *K*. *veneficum* host used in this experiment was CCMP 1975 isolated from the Chesapeake Bay which differs in toxin profiles and ITS sequence from *K*. *veneficum* isolated from the north Atlantic that was used for chloroplast genome sequencing [43]. The combination of AT bias, sequence size over 1 kb, over ten fold coverage, and matches between transcriptome and genome assembly could be used simultaneously to define genuine parasite genomic sequence.

### Anomalous alignment results

**Fig 1:**
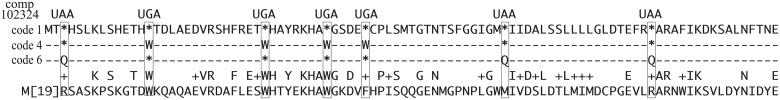
A sequence translated using three different genetic codes illustrating changes in BLASTx scores. The nucleotide sequence from comp102324 was translated as a query under three different genetic codes with the dashes (-) representing identically translated codons. The top amino acid translation uses NCBI genetic code 1, where UGA, UAA, and UAG are all interpreted as stop (*). These codons are shown above the amino acid translation and are boxed. The NCBI genetic code 4 translates UGA as tryptophan (W), increasing the number of pairwise identities in this example by three, and the number of positive matches by one (identified in the midline with +). However, three UAA stop codons still interrupt the open reading frame. The third translation using NCBI code 6 interprets UAA and UAG as glutamine (Q), which does not increase the number of pairwise identities, but does increase the number of positives, or similar amino acids by two. In this example there are no UAG codons. The top blast hit (*Candida tenuis* ATCC10573 gi 575519749) and pairwise identities or positives when translating all three stop codons as amino acids are shown below the different translations.

The BLASTx program reported the number of identities and positives in the pairwise alignment between each translated query and the best subject sequence or top hit. Identities are the count of identical amino acids in the alignment, while the positives are the identities plus all the amino acids with positive scores based on the amino acid scoring matrix (BLOSUM 62). Some BLASTx pairwise amino acid alignments of AT rich sequences contained stop codons when using the default or standard genetic code (NCBI genetic code 1) (Fig 1). The standard genetic code treats UGA, UAA, and UAG as stop codons. In the example shown in Fig 1, interpreting UGA as tryptophan (NCBI genetic code 4) increased the alignment score by three identities and one positive. However, translating UGA as tryptophan did not resolve all the stop codons. Translating UAA and UAG as glutamine (NCBI genetic code 6) increased the positive score by two. One UAA codon, when translated as glutamine was aligned with a methionine, not increasing the score, but removing the in frame stop codon. Thus, in sequences where all three typical stop codons were present in the BLASTx alignment, the union of the identity or positive scores under the two alternative codes yielded higher scores than either alone. However, in the example shown in Fig 1 the increased score accounts for most but not all stop codons. No single genetic code currently implemented in BLASTx simultaneously translates UGA as tryptophan, and UAA and UAG as glutamine, because then a stop codon would not be specified.

**Fig 2:**
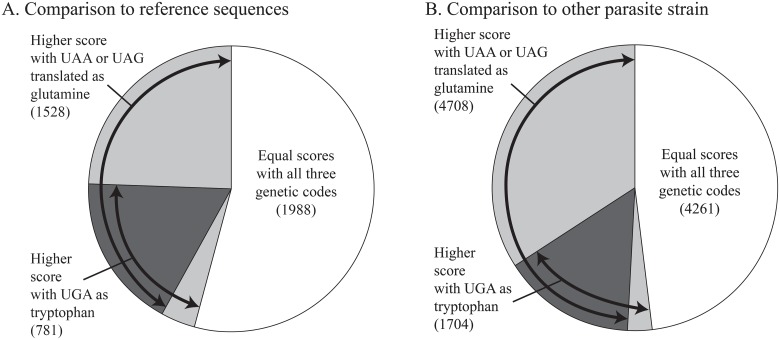
Proportions of BLASTx hits producing different raw pairwise scores when translated with three genetic codes. A. BLAST using the high AT content, putative parasite, sequences as queries and the NCBI reference sequence database as the subject (3,652 hits) or the translated transcriptome data from *Amoebophrya* sp. ex *Akashiwo sanguinea* as the subject (9,589 hits). For the comparison with the reference sequence database 3,652 queries with e-values ≤10^−10^ roughly half (unshaded portion) had equal scores with all three genetic codes. Of the remaining sequences, most contained UAA or UAG codons which, when translated as glutamine increased the BLASTx score (light grey shading). Many of these sequences also contained UGA with increased scores when translated as tryptophan (darkest shading contains both UGA and UAA or UAG). A small fraction of the UGA-containing sequences did not have increased scores when UAA or UAG were translated as glutamine (light grey shading).

The identity and positive scores for top hits were compared between the three genetic codes for all 3,652 unique putative parasite sequences with BLASTx alignments in the initial annotation (Fig 2A). These 3,652 unique sequences were reduced from a total of 4,908 sequences when additional sequence variants were included, and all of the proportions are calculated without including sequences from the same graph component. Over half (54%) of putative parasite sequences had equal scores under the standard code and both alternative codes. When using NCBI genetic code 4 (UGA as tryptophan), 781 sequences had a higher number of identities or positives when compared to the NCBI reference sequence database. Similarly, when using NCBI genetic code 6 (UAA and UAG as glutamine) 1,528 sequences had higher number of identities or positive matches than the standard genetic code (Fig 2). Most queries with better scores when UGA was coded as tryptophan also had better scores when UAA and UAG were coded as glutamine (Fig 2). The increase in the number of identities for a given sequence ranged from one to 38, and the increase in the number of positive matches ranged from one to 51 when the *de novo* assembly was compared to the reference sequence database.

The proportion of hits with equal and increased scores under different genetic codes was slightly larger in the strain to strain comparison than the proportion when using the reference sequence database (Fig 2B). When putative parasite sequences from *Amoebophrya* sp. ex *K*. *veneficum* were compared with the combined *Amoebophrya* sp. ex *A*. *sanguinea* host and parasite dataset formatted as a database there were 9,589 novel hits at an e-value cut-off ≤ 1e-10 to (Fig 2B). For the *Amoebophrya* sp. ex *K*. *veneficum* sequences that had hits to the reference sequence database, 4,761 (of the 4,908 with redundancy) sequences also had BLASTx results of 1e-10 or lower in the comparison between strains. Overall, across the BLASTx comparisons between the two *Amoebophrya* transcriptomes, there were 5,024 instances of UGA that, when translated as tryptophan, increased the alignment score.

StringTie was used to infer transcripts from the SPAdes assembly using the RNAseq reads. StringTie transcripts annotated with Diamond as a rapid BLASTx tool against the non redundant database recapitulated results from *de novo* assembly of RNAseq reads. A total of 31,094 transcripts were inferred by StringTie and between 4,190 (genetic code 1) and 4,408 (genetic code 6) had hits to the non redundant database with an e-value cut-off <1e-9. As with the *de novo* assembled transcripts, the UAA and UAG codons were more frequent than UGA– 38,025 were counted in the top hits, while UGA codons were found 9,369 times.

As a sort of control three datasets were used as queries: 1) <58% AT content, or putative host fraction from the same RNAseq data as the parasite sequences, 2) the ≥58% AT content parasite sequences, and 3) the combined *Amoebophrya* sp. ex *A*. *sanguinea* host and parasite transcriptome assembly. These three datasets were compared to a database of a single species, *Perkinsus marinus* (which is part of the reference sequence database) using different genetic codes as described above. Using a single species database provides more consistency in the searches and speeds the analysis. For the first dataset, the <58% AT content sequences attributed to *K*. *veneficum* where each query was translated with multiple codes as above, there were 219 instances where in frame stops were found out of 13,314 top BLASTx matches with an e-value cut-off of <1•10^−10^ and 83 examples of increased BLASTx scores (host organelle transcripts are AT biased and treated below). Of these 83 sequences with increased BLASTx scores eleven were likely parasite sequences based on genomic coverage and near 58% AT content, leaving a total of 72 out of 13,314 total hits with increased scores in this pairwise comparison. When comparing the second dataset of 4,906 sequences that had matches to the reference sequence database with ≥58% AT content fraction to *P*. *marinus* there were 3,480 BLASTx results of which 1,761 had stop codons in the query sequences and 1,402 instances where either one or both alternative code produced better results than the standard genetic code. Finally, in the third dataset of combined *Amoebophrya* sp. ex *A*. *sanguinea* host and parasite sequences there were 18,757 queries with e-values <1•10^−10^ when compared to *P*. *marinus* (top hits only) and 377 instances of stop codons were seen in these queries. Of these, only 158 had increased scores with alternative genetic codes, of which 81 matched a single sequence from *P*. *marinus*, XP_002768980. As a test for a non-translated RNA misannotated as protein coding, the corresponding nucleotide sequence from *P*. *marinus* (XM_002768934) was used for an RFAM search and BLASTn comparison with *Amoebophrya* sp. ex *A*. *sanguinea*, neither of which revealed conserved nucleotide matches. For sequences likely derived from the *K*. *veneficum* host or the combined *Amoebophrya* sp. ex *A*. *sanguinea* sequences there were infrequent examples of increased scores in BLASTx comparisons with different genetic codes.

### Identifying the amino acids associated with typical stop codons

**Fig 3:**
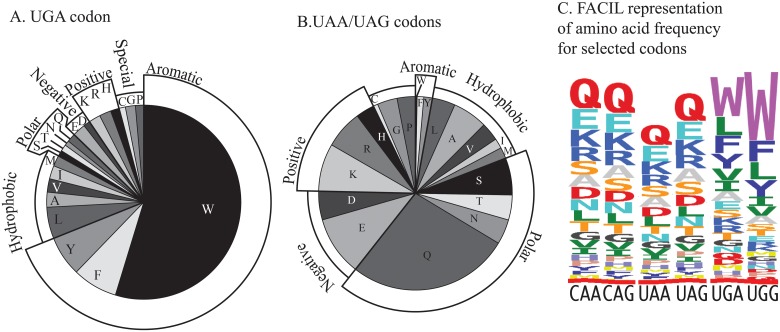
The amino acids aligned to UGA, UAA and UAG. A. The amino acids found in the top BLASTx alignments to the NCBI reference sequence protein database where scores were increased relative to the standard genetic code when encoding UGA as tryptophan. The amino acids are grouped into hydrophobic, polar, negative or positively charged, and special cases. The UGA commonly was aligned to gaps, but these cases are not shown here. Only alignments to tryptophan, tyrosine, or phenylalanine increase BLASTx positive alignment scores with the matrix used; however all UGA codons within sequences with increased BLASTx scores are used in this comparison. B. Amino acids associated with UAA and UAG codons in BLASTx comparisons where scores were increased when translating UAA and UAG as glutamine. Gaps were not included in this analysis. C. FACIL analysis of the genetic code. The four possible glutamine and two likely tryptophan codons are shown. The most commonly found amino acids when these codons are translated and compared to the protein family (pfam) database are shown as a sequence logo with height proportional to frequency as inferred by FACIL.

In *Amoebophrya* sp. ex. *K*. *veneficum* sequences with increased BLASTx scores against the reference sequence database using alternative genetic codes, the most common amino acids aligned with query UGA codons were tryptophan, leucine, tyrosine, and phenylalanine (Fig 3A). Although increased scores indicated at least one site where UGA as tryptophan increased the pairwise alignment score, the results described in Fig 3 included all alignment positions where a UGA was present, including polar amino acids but not gaps. The default BLASTx BLOSUM62 scoring matrix for tryptophan has only phenylalanine, and tyrosine as positive matches; selenocysteine was very infrequent and is not represented (see below). For UAA and UAG codons the most frequent amino acid was glutamine, but glutamate, lysine and arginine were also present and would be counted as positive matches (Fig 3B). The results from FACIL were consistent; UAA and UAG were frequently associated with glutamine or glutamate (Fig 3C). The FACIL results for the standard glutamine codons, CAA and CAG were similar to those for UAA and UAG. With FACIL analysis UGA and UGG were associated with trypophan, leucine, tyrosine, and phenylalanine. As a further test, the proportion of UGA aligning with tryptophan in comparisons between the two parasite strains were calculated for alignments with e-value cut-offs from <1•10^−10^ to <1•10^−200^ using the set of putative parasite genes with increased scores when compared with the reference sequence database. The proportion of tryptophan for UGA codons increased from 72 to 83% as the e-value stringency was increased. A similar test with UAA or UAG codons showed 42% matching glutamine at an e-value cut-off of 1•10^−10^ increasing to 53% at an e-value cut-off of 1•10^−200^.

A comparison using all available genetic codes in BLASTx was conducted against the *P*. *marinus* database using the 2,006 queries from *Amoebophrya* sp. ex *K*. *veneficum* with increased scores against the reference sequence database when using alternative genetic codes 4 and 6. In this comparison of 19 genetic codes, the highest positive score was found in 1,020 of 1,499 hits when using NCBI code 6 (UAA and UAG as glutamine), followed by genetic code 1 with 251, while 507 sequences had no hit (at an e-value cut-off of 1•10^−10^). Recoding UGA as tryptophan is common to nine of the 19 different genetic codes tested.

### Determining the stop codon

**Fig 4:**
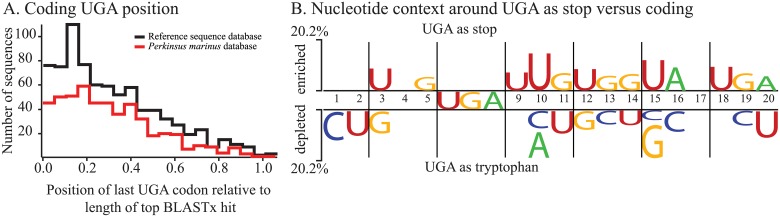
Position and nucleotide context around UGA codons. A. The amino acid position of the last or most 3’ UGA codon in the query sequence alignment was divided by the total length of the top hit in the BLASTx comparison and plotted as a histogram to indicate where the UGA codons were found relative to the total length of the subject sequence. Comparisons to *P*. *marinus* (red) and the reference sequence (black) databases are shown for AT biased sequences with increased BLASTx scores when using alternative genetic codes. **B**. The composition before and after UGA apparently encoding stop versus UGA as tryptophan, based on increased BLASTx scores when UGA was translated as tryptophan. In frame UGA as stop for 457 non-redundant sequences from the parasite fraction were compared to 333 in UGA as tryptophan using two sample logo with the t-test. The size of the nucleotide shows the relative enrichment or depletion of each nucleotide in the upstream and downstream positions, but is shown only for nucleotides enriched or depleted at a p-value below 0.05 (see S2 Fig for raw frequency values).

Because increases in BLASTx scores were observed for all three typical stop codons, the stop codon was determined from a set of highly conserved sequences. Of the 158 putative parasite sequences in the *de novo* transcriptome assembly with a zero e-value to NCBI reference sequences, 74 had a complete open reading frame ending in a UGA codon that was in a similar position to the stop codon of the best BLASTx result from the reference sequence database. Five contained upstream in frame UGA codons, followed by a UGA codon in a position consistent with a stop, again based on comparison to top blast hits. One had an apparent intron, but also contained a UGA consistent with a stop codon. The stop codon was predicted to be UGA in all cases and no UAA or UAG codons were found in positions consistent with a stop. The remaining 78 sequences were partial in comparison to the BLAST subjects and were not treated further. Similarly the stop codon was consistently UGA in ribosomal proteins aligned for phylogenetic analysis [7]. In contrast, the position of UGA codons inferred to be tryptophan based on BLASTx comparisons were biased towards the start or 5’ end of mRNA when compared with the total length of the top hit (Fig 4A). This result was consistent when using comparisons to either the reference sequence database or *P*. *marinus*.

The nucleotide frequency before and after UGA codons differed only slightly between those inferred to encode tryptophan or as stop (Fig 4B). For UGA codons that likely encode tryptophan based on increased BLASTx scores there was a repeated pattern of increased AT bias at third positions both before and after UGA codons (S2 Fig). This would be expected in coding sequences where second and first positions are more constrained, but was not seen before or after stop codons. The two sample logo program uses a t-test to determine significant enrichment or depletion at p values less than 0.05 for the bases centered around the two different senses of the UGA codon (Fig 4B). This test reveals a general pattern of increased U two and seven bases downstream of the stop codon. However these results need to be interpreted with caution as the overall abundance of U was below 20.2% at these positions. Inverting the input order to demonstrate enrichment or depletion in coding sequence relative to stop codons showed even lower enrichment values of 15.2% for significantly enriched or depleted bases.

### Sources of interrupted reading frames: Organellar genes, RNA editing, and selenocysteine

Organellar transcripts, RNA editing and UGA codons encoding selenocysteine could independently contribute to finding typical stop codons in transcriptome datasets. For example, host organelle transcripts were found in the ≥58% AT content sequences. A total of 26 transcript sequences (21 non-redundant) with AT content from 59–66%, had >90% nucleotide identity to the previously determined *K*. *veneficum* chloroplast sequences [33]. Only one sequence had an increased score with the alternative genetic code, and that fragment corresponded to a chloroplast rRNA region.

The mitochondrial genome of dinoflagellates and apicomplexans has only three protein coding genes, coxI, coxIII, and cytB [44]. RNA editing is also a common feature in dinoflagellate mitochondrial transcripts and provides an opportunity to test for RNA editing as a potential reason for in-frame stop codons in *Amoebophrya* sp. ex *K*. *veneficum* transcripts. Because these protein-coding genes are conserved, AT biased, and highly expressed, they can be readily identified based on text searches of annotations followed by manual inspection and sorted between host and parasite using BLASTn searches against the uninfected host data [32,34]. Comparing genomic and expressed sequences, the mitochondrion-encoded transcripts from *Amoebophrya* sp. ex *K*. *veneficum* showed changes consistent with mitochondrial editing (Table 1). The three parasite protein-coding transcripts from the mitochondrion for coxI, coxIII, and cytB, were strongly AT biased (68–69% AT content). Comparing the genome and RNA-seq data for these transcripts showed a total of 57 differences, similar to values seen in core dinoflagellates [44] and *Hematodinium* sp. [45]. However, in *Amoebophrya* sp. ex *K*. *veneficum* the editing was limited exclusively to A->G and T->C changes.

**Table 1:** Differences between the genomic and expressed versions of mitochondrial genes found in *Amoebophrya* sp. ex *Karlodinium veneficum*.

Gene names	# edited sites	A->G	T->C
coxI	21	16	5
coxIII	21	10	11
cytB	15	14	1

Similarly, the rare amino acid selenocysteine is typically encoded by UGA codons that are contingently translated based on features of the 3’ UTR [46]. Only two instances of selenocysteine were found in the BLASTx search, however, automatically generated open reading frames may not properly map UGA to selenocysteine, instead treating this codon as a stop. A previous survey of *Oxyrrhis marina* found a total of four selenoproteins [47]. Based on text searches of annotations and comparisons to core dinoflagellate transcriptomes, for the AT biased, putative parasite sequences a total of three likely selenocysteine-containing genes were found: selT, selM, and selO, each with one UGA likely encoding selenocysteine. Using SECISaln [46], a selenocysteine insertion sequence was found in the 3’ UTR of selT.

### Gene ontology terms

The putative parasite sequences from *Amoebophrya* sp. ex *K*. *veneficum* were divided into two bins based on the optimal BLASTx score when using reference sequence database. The first contained the 2,900 (1,988 non redundant) sequences where the scores were unchanged with different genetic codes (standard set), and a second with 2,006 (1,664 non redundant) sequences where the optimal score was found using either the ciliate or mitochondrial genetic codes or both (alternative set) (Fig 2A). These same two sets of sequences were also used for codonw analysis as described in detail below. The overlap between sequences containing UAA or UAG and those with UGA that increased BLASTx scores demonstrated these codons were often detected together on the same transcripts (Fig 1). Because translation with no defined stop codon is very difficult to accomplish *in silico* especially with long UTR, a relational strategy was used to create proxy amino acid sequences for GO analysis. Nucleotide sequences from *Amoebophrya* sp. ex *K*. *veneficum* were matched to translated sequences from *Amoebophrya* sp. ex *A*. *sanguinea* based on top BLASTx hits. The top matching *Amoebophrya* sp. ex *A*. *sanguinea* protein sequences were extracted and then sorted into those that contained typical stop codons in *Amoebophrya* sp. ex *K*. *veneficum* (1,118 sequences with average length of 1,059 amino acids) and those that did not (1,125 sequences with an average length of 629 amino acids), followed by annotation using BLAST2GO. The 1,118 sequences with the alternative codons had 359 KO terms and were enriched (p ≤ 0.05) for microtubule motor function, ion channel activity, and ATP and DNA binding (S3 Fig). For example, most of the dynein heavy chain protein family members (described in detail below), and all three DNA dependent RNA polymerase large subunits I, II, and III demonstrated alternative codon use. The coding sequence most enriched for UGA codons contained 12 UGA codons interspersed with 13 UGG codons and had a blast2GO hit to DNA phytolyase. On the other hand, the standard set of 1,125 sequences with 717 KO terms was enriched for translation initiation, ribosomal proteins, cation transporters, unfolded protein binding, and threonine type endopeptidase activity (S3 Fig).

### Typical stop codons in dynein heavy chain transcripts

With a genome and transcriptome survey in hand and preliminary results from GO terms suggesting potential for codon bias and different categories of transcripts, a tractable gene family was selected which would provide a robust and practical challenge for the assertion that UGA was conditionally translated and often linked with the other two typical stop codons. Another goal was to test if pseudogenes or unspliced introns led to the BLASTx results presented above. Dynein Heavy Chain (DHC) was used as an example since these are amongst the longest transcripts and inference of the 13 to 16 kb transcripts required long sections of genomic assembly. Preliminary analysis of the *de novo* assembled transcripts suggested high expression levels and a wide range of typical stop codons for sequences annotated as dynein heavy chain. From an annotation perspective the gene family is tractable, has a well organized nomenclature, and there are different roles for the different subclasses [48]. Using 67 queries for *Symbiodinium kawagutii* gleaned from the CyMoBase website and a cutoff of >3,000 pairwise amino acid alignment to cover the majority of the query sequences, a total of 14 DHC transcripts were found in tBLASTx searches against the StringTie transcripts (Table 2). The identity between *S*. *kawagutii* and *Amoebophrya* sp. ex *K*. *veneficum* ranged from 46% over 3,680 amino acids for DHC1, to 67% over 4,717 amino acids for DHC3A. Using the nomenclature of Kollmar [48] and best tBLASTx identity for preliminary orthology assessment, the inventory putatively identified DHC subtypes 1 to 9. Reciprocal best hits between genomes are not yet tractable, as both the *S*. *kawagutii* and *Amoebophrya* genomes are likely not complete. For these dynein sequences, the 3’UTR varied from 365 to 1,807 bases, with an average length of 1,022 bases.

**Table 2:** Comparison of Dynein Heavy Chain (DHC) sequences from *Symbiodinium kawagutii* as queries to *Amoebophrya* sp. ex *Karlodinium veneficum* StringTie transcripts.

Name	Transcript label	% identity	Alignment length (AA)	Query alignment end (AA)	Query length (AA)	Subject alignment end (ntd)	Subject length (ntd)	Introns	UGA as W	UAR as Q	3' UTR (ntd)
DHC1	STRG.3119.1	46.22	3,680	4,455	4,572	11,615	12,382	7	18	84	365
DHC2	STRG.16568.1	49.95	4,190	4,295	4,296	12,612	13,180	5	7	150	565
DHC3A/B	STRG.14215.1	67.06	4,717	4,642	4,642	14,581	15,988	12	0	1	1,407
DHC3A/B	STRG.12749.1	66.59	3,798	4,642	4,642	15,457	16,782	11	0	24	1,325
DHC4A	STRG.8604.1	62.41	4,206	4,174	4,174	13,217	15,024	7	0	0	1,807
DHC4A	STRG.7562.1	60.92	4,204	4,174	4,174	12,514	13,177	5	0	5	663
DHC5	STRG.7782.1	65.94	3,693	4,602	4,602	14,964	15,924	13	3	44	960
DHC6	STRG.5796.1	58.25	4,651	4,520	4,520	14,285	15,070	7	5	58	776
DHC7A/B	STRG.7345.1	56.61	5,202	5,022	5,022	15,830	17,637	10	0	0	1,807
DHC7C	STRG.10146.1	50.78	3,675	3,985	3,985	16,335	17,265	7	2	49	930
DHC8A/B	STRG.7225.1	56.51	4,116	4,243	4,244	13,281	14,375	8	1	40	1,091
DHC8C	STRG.12802.1	55.15	3,706	4,196	4,197	13,706	14,602	8	3	46	890
DHC9A	STRG.885.1	57.05	4,058	4,063	4,063	13,424	14,426	9	4	60	1,002
DHC9B/C	STRG.15239.1	52.67	4,171	4,066	4,066	13,560	14,289	10	3	99	729

**Fig 5:**
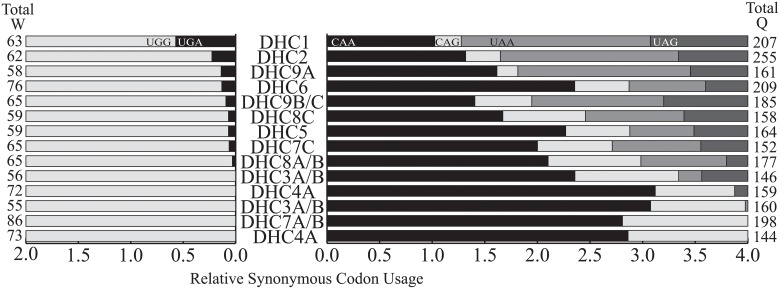
Tryptophan and glutamine codons in the dynein heavy chain alignments. The relative synonymous codon usage based on UGG and UGA as the two codons for tryptophan (W) on the left and the four likely glutamine (Q) codons CAA, CAG, UAA, and UAG on the right were inferred from aligned regions of Dynein Heavy Chain (DHC) family members (as shown in Table 2) based on top tBLASTx hits. The total number of tryptophan or glutamine residues in the aligned regions is shown at the end of the bars for each gene.

On the extremes, the 5,202 amino acid alignment for DHC7A/B contained no typical stop codons, while DHC2 had 157. After extracting the in frame nucleotide region corresponding to the best tBLASTx alignment the two likely tryptophan (UGG and UGA) and four likely glutamine codons (CAA, CAG, UAA, and UAG) were counted and relative synonymous codon usage (RSCU) values calculated [49] (Fig 5). Tryptophan was found between 55 and 86 times and glutamine was two to three times more frequent with 144 to 255 occurrences in these alignments. The UGG codon was strongly favored over UGA across all fourteen alignments. Glutamine codons were more variable, in nine DHC alignments CAA was the dominant codon (RSCU values of 3.12 to 1.67 out of 4). However, in three cases (DHC1, DHC2, DHC9A) UAA codons were most frequent with RCSU values of 1.80 to 1.64. There was a general correlation between high UAA usage and UGA, while alignments with fewer UGA codons contained more CAA and CAG codons.

### Genomic tRNA compared with mRNA codons

The genome survey of *Amoebophrya* sp. ex *K*. *veneficum* contained 316 likely parasite genome-encoded tRNAs. This total included 22 pseudogenes based on tRNAscan-SE (S1 Spreadsheet for complete list) [39]. The tRNA were considered to be genuine parasite sequences based on the following criteria: no BLASTn hit to the host chloroplast or *Kordia* sp. bacterial contaminant, assembly by both ABySS and SPAdes, and a SPAdes coverage at least 10. For simplicity, the following text refers to the mRNA codon rather than the tRNA anticodon. Only one tRNA was found for UGA, and identified as a selenocysteine tRNA (see S4 Fig for proposed secondary structure). In addition, one UAA and two UAG tRNA were found. The UAA tRNA scan score was 53.59 with a histidine isoform score of 64.7. and a glutamine isoform score was 62.6. The same genome contig also contained a predicted UAG tRNA with a score of 53.18 and best match at 65.4 to the histidine isoform versus 61.2 for the glutamine isoforms. Finally the second UAG had a tRNA scan score of 49.44 and with optimal isoform scores of 67.0 for glutamine and 49.4 for histidine (see S4 Fig for proposed secondary structures).

**Fig 6:**
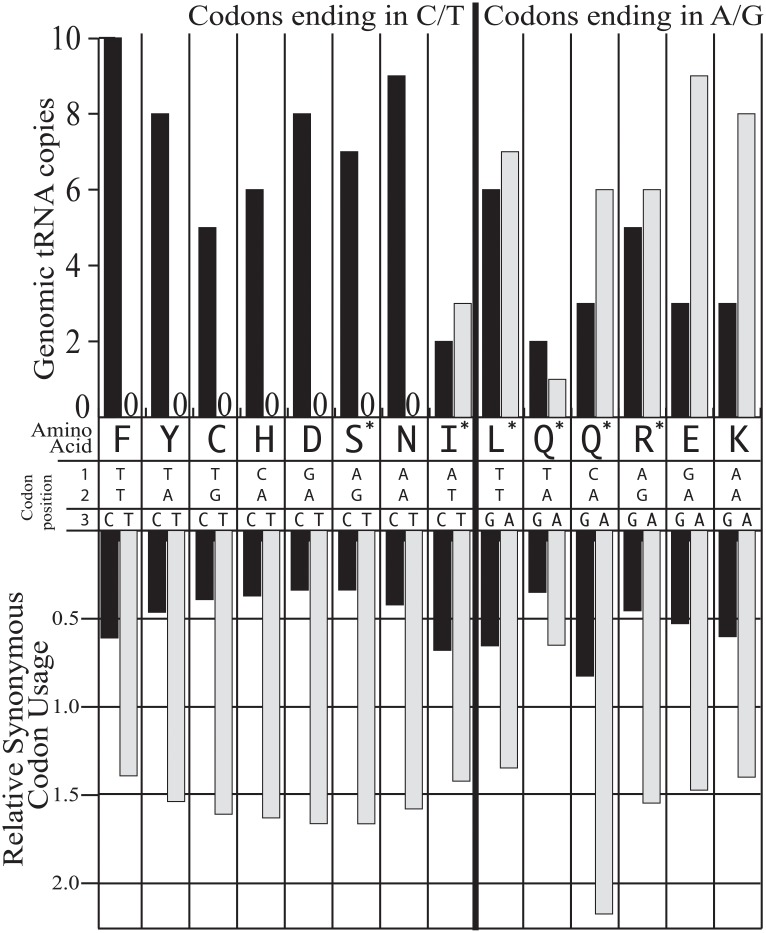
Comparison of codons in transcripts versus tRNA count in genomic assembly. The relative synonymous codon usage (RSCU) for 28 codons in 1,466 transcripts are shown as a bar graph in the bottom portion, while the top portion shows number of complementary tRNA found in the genome survey. The black bars are used for codons ending in G or C, while the grey bars are for codons ending in A or U. The codons were sorted based on third positions ending in C or U (left portion of figure) when compared with those ending in G or A (right portion). Due to redundancy in the genetic code some amino acids have between one and six possible codons. The 28 codons shown include the 16 third position informative codons ending in C or T and A or G, or amino acids with just two codons. For comparison an additional 12 codons for amino acids with three to six codons are shown and indicated with an asterisk. The RSCU values for likely glutamine codons (UAG, UAA and CAU, CAG) sum to four while phenylalanine with two codons would sum to two and serine to six. All of the codons that were synonymous at third positions and AUG, AUA, UGG and UGA were excluded.

There was bias against tRNAs with bases complementary to third codon position U (Fig 6). The amino acids phenylalanine, tyrosine, cysteine, histidine, asparagine, and aspartate have two codons, one ending in C, the other in U. For all six of these amino acids at least 5 tRNA were found complementary to codons ending in C, and none ending with U. Although serine has six possible codons, the serine codons AGC or AGU showed none ending in U and 7 with C. Overall, of the 16 codons ending in U, complimentary tRNA were not found for 8. The only other codons missing from the tRNA pool were UCC and non-selenocysteine UGA. However, for codons ending in A or G, there was not a strong pattern in the annotated tRNA, except in the case of UGG versus UGA, where 10 tRNA fully complementary to UGG were found and none for UGA, again except for the selenocysteine tRNA.

The codon analysis using codonw required a choice between using the NCBI genetic code 6 or 4; even with a custom genetic code, UGA would still be contingently translated as tryptophan and then stop. Here NCBI code 6 was chosen, since UAA and UAG codons were not translated ambiguously and were more common than UGA (Figs 2 and 5). The starting set was the 3,652 parasite sequences with BLASTx e-values ≤ 1e-10 that were translated using longorfs. Longorfs translated 1,466 sequences containing 1,114,081 codons. Dividing the data into two sets, one with increased scores with alternative genetic codes (558 orfs with 434,300 codons) and the second without (908 orfs with 706,781 codons), showed similar length and GC content, but an increase in the number of codons from 46 to 48, as would be expected when UAA or UAG are used (S1 Spreadsheet for full codonw output).

In strong contrast to the genomic tRNA bias against codons ending in U, the transcripts from *Amoebophrya* sp. ex *K*. *veneficum* were more AT biased in the third codon position than the average across all positions (Fig 6). The third position synonymous, or 3s, values from codonw showed GC3s was 0.26, T3s was 0.49, and A3s was 0.44, in all cases demonstrating bias for A or U in the third position of synonymous codons (S1 Spreadsheet). Codon usage for amino acids with only two codons ending with C or U, such as phenylalanine, tyrosine, cysteine and others, was strongly biased for codons ending in U (Fig 6). Similarly amino acids with two possible codons ending in A or G, such as glutamate and lysine, preferred codons ending in A. This bias partially extended to the four codons likely encoding glutamine, where codons ending in A were strongly favored over codons ending in G. However, first position C (CAA and CAG) was favored over first position U (UAA or UAG), in contrast to the general AT bias. However, because UGA and UAA or UAG codons often occur together as shown in Figs 1 and 5, the analysis likely underestimates the codon usage values for UAA and UAG, since sequences with detectable coding UGA were excluded from this analysis.

## Discussion

The three typical stop codons UAA, UAG, and UGA likely encode amino acids in *Amoebophrya* sp. ex *Karlodinium veneficum*. The data presented here relies on alignment between sequences to infer the genetic code, a frequently used strategy that predates BLAST [18,19,22,25,26,50]. An advantage of using BLASTx to create the alignments is rapid assessment, revealing unusual codon translations across an entire dataset. However, the strategy likely underestimates the abundance of such codons because even when compared with the relatively closely related *Amoebophrya* sp. ex *Akashiwo sanguinea*, a large fraction of sequences do not have BLAST alignments between strains. Roughly half the parasite sequences with BLASTx matches have in frame stop codons when translated with the standard genetic code and increased alignment scores when using genetic codes 4 or 6. The proportion of sequences with typical stop codons and those without remained similar across multiple different subject databases, including in comparisons between the strains and when using the genome-inferred or the *de novo* assembled transcripts as queries.

In this dataset, despite only one fifth of BLASTx matches showing increased scores when UGA was translated as tryptophan, the aggregate number of individual mRNAs with increased scores was near one thousand, and the total number of such codons was over 5,000 in the most comprehensive comparison with the other parasite strain. For the more frequent amino acid glutamine, the aggregate totals were substantially higher (Figs 2 and 3). These values likely represent a lower bound as gaps or amino acids without identity or a positive score would not contribute to alignment score increases but could still contain typical stop codons. For example in Fig 1, of the seven stop codons, only six would be counted by changes in scores (Fig 1). Thus, the total number of typical stop codons that encode amino acids is likely underestimated using this method, because increased scores only occur when the relevant codon is aligned with an identical or similar amino acid, not when gaps or dissimilar amino acids were aligned. However, the strategy also removes several potential artifacts. For example, introns would introduce alignment gaps, rather than increase the alignment score, and introns are likely to interrupt the open reading frame and thus break the BLASTx alignment.

There are several other possible reasons to find typical stop codons in transcriptome datasets. For example, RNA editing, where specific bases are edited after transcription could explain at least some typical stop codons. In the current dataset RNA editing was readily observed between genomic and expressed sequences for the three protein coding genes encoded in the mitochondrial genome, however no cases of editing were observed for nuclear encoded mRNA. Similarly, incorrectly annotated rRNA sequences or selenocysteine codons explain only a handful of the UGA codons.

Invoking sequencing error seems unlikely as the host sequences only rarely contained stop codons. When comparing results from the putative host fraction only a small fraction contained in frame stop codons in their BLAST query sequences, and an even smaller fraction resulted in increases in the alignment score. Similarly, the data from the other strain, *Amoebophrya* sp. ex *Akashiwo sanguinea* provides another reference for how many typical stop codons might be found by chance in BLASTx comparisons (~2%, with fewer causing increased alignment scores). However, it is important to note that both the host data, selected based on AT bias, and the data for the other parasite strain are both less AT biased than sequences from *Amoebophrya* sp. ex *K*. *veneficum*, so the overall frequency of UAA would be lower. Overall bulk quantity and frequent increases in alignment scores suggest the typical stop codons are coding in *Amoebophrya* sp. ex *K*. *veneficum*.

### The amino acids likely encoded by UAA, UAG and UGA

The identity of the amino acids and the optimal genetic code were initially examined using a heuristic measure as demonstrated by Fig 1. Pairwise alignments often revealed the relatively rare and often conserved amino acid tryptophan across from UGA codons, while frequently the amino acid glutamine or amino acids with similar properties were found across from UAA or UAG codons. Similarly, the FACIL program reported results congruent with translating UGA as tryptophan and UAG or UAA as glutamine. This was verified by using all the available genetic codes in BLASTx searches and the ciliate genetic code provided the largest number of optimal scores. There are a number of potentially confounding effects that could be at work. For example the BLOSUM matrix accounts for amino acid substitutions but tryptophan and glutamine frequencies vary (Fig 5), and the ideal genetic code may not yet be available in BLAST. Thus, the exact identity of the amino acid encoded by these codons has not been experimentally verified, even if the results currently suggest that UGA encodes tryptophan and UAA and UAG encode glutamine.

The translation of UAA and UAG as glutamine is a broadly distributed alternative genetic code, so far described from multiple lineages including ciliates, green algae, diplomonads, *Amoeboaphelidium protococcarum*, and oxymonads [16,18,19,51]. The recoding of UAA and UAG was in some cases correlated with AT bias, which in turn reflects selection–either a result or cause of changes in the genetic code. Here, AT bias is used as the defining feature of parasite sequences, yet the typical stop codons were only observed in about half the annotated sequences. In *Tetrahymena thermophila* and *Paramecium tetraurelia*, ciliates with more extreme AT bias, UAA is the most frequent glutamine codon [52,53]. In *Amoebophrya* sp. ex. *K*. *veneficum* the most frequently used glutamine codon was CAA, followed by CAG, then UAA (Figs 5 and 6). However, the UAA or UAG usage estimate may have been confounded because sequences with UGA codons were not used in that analysis and are often linked as shown in the DHC family analysis. Accurately predicting the CDS when UGA is conditionally translated is not possible, so sequences with UGA were excluded during translation for bulk codon usage estimates.

Recoding UGA as tryptophan has been frequently observed in organellar genomes from different eukaryotic lineages (as well as bacteria) [12,22,54] and has also been reported from eukaryotic nuclear mRNAs [16,24–26]. In addition, readthrough of stop codons is probably more frequent than previously recognized [55]. Unlike readthrough of UGA, in the case presented here, UGA is less frequent at the 3’ ends of mRNA (Fig 4), and the codon often occurs multiple times in a single mRNA (Figs 1 and 5, Table 2). Recently other examples of all three typical stops being recoded as amino acids were seen in ciliates [24,25]. Stop codon reassignment and contingent translation of UAA or UAG as amino acids or stop in the trypanosomatid *Blastocrithidia* sets a precedent for the results shown here with inferred contingent translation of UGA as stop and tryptophan [26]. The inferred genetic code for *Amoebophrya* sp. ex *K*. *veneficum* seems to exactly match that seen in the ciliate *Parduczia* sp. [24]. However, in the ciliate examples the 3’ UTRs were only about 20 bases, much shorter than those found here for *Amoebophrya* (Table 2). In ciliates stop codon prediction may follow a simple rule–those that are near the end of mRNA are likely acting as stop, while in other sites the ribosome continues translation.

### Correlation between UGA and UAA or UAG codons in transcripts

In the *Amoebophrya* sp ex *K*. *veneficum* sequences the strong correlation between UGA codons and UAA or UAG codons, such that UGA codons that increased alignment scores in the absence of increased scores for UAA or UAG codons were rare (Figs 2 and 5). In other words, the three codons frequently occur together in transcripts. On the other hand, these codons are completely absent from about half of annotated transcripts, or at least do not increase the alignment scores in these cases. These results suggest the potential for two sets of transcripts. The first contains UGA and UAG or UAA codons while the second lacks them. By selecting only categories with relatively deep annotation of GO terms, there were biases between mRNA with and without the observed alternative codons (S3 Fig). The GO terms associated with translation, including the abundantly expressed, readily annotated, ribosomal proteins lack alternative codons. Other functions such as DNA binding or microtubule motor activity are enriched with typical stop codons. Oxidoreductase activity and ATP binding were more balanced, with many terms found in both gene sets. The GO assignments are biased by database content, annotation of more conserved genes, and the ontology terms themselves. This is by no means a conclusive demonstration of links between the function and codon bias, but suggests the typical stop codons are not randomly distributed across transcripts.

The DHC results confirmed an uneven distribution of these codons and reinforced the pattern seen with GO terms. First of all, the DHC results addressed the possibility of duplication into a pseudogene containing stop codons while a functional copy without stop codons was maintained. In this parasite, duplicated DHC genes with and without typical stop codons were not seen. The analysis also did not show RNA editing and validated inferred transcripts from the genome. This more detailed analysis suggests a more nuanced view than the binary results from BLASTx and GO term analysis. In the DHC example, two transcripts have no typical stops, some have a few, others between twenty and sixty, while others are have more than one hundred typical stop codons such as DHC2, DHC9B and DHC1 (Table 2, Fig 5). The DHC proteins are mechanistically similar, and generally similar in length, but have different cellular roles [48]. Extrapolating, DHC1 and DHC2 are cytoplasmic with many stop codons, while DHC4A is an outer axonemal and DHC7A/B an inner axonemal heavy chain with no stop codons [48]. Even if the specific roles of the different DHC family members in *Amoebophrya* are not yet clear, the results for this gene family demonstrate that typical stop codons are not uniformly distributed across different transcripts. This may impact expression as efficient stop codon translation may happen during specific life stages, cellular compartments, or growth conditions. The clear distinctions between a feeding, division, and infective life stage in the parasite could be linked with changes in translation and the unusual genetic code. Previous studies of differential expression suggest a large scale transcriptome remodeling during the infection cycle in other *Amoebophrya* strains [56,57].

### Genomic tRNA survey

There was also a very strong contrast between the frequency in transcripts and number of genome-encoded tRNA for codons ending in U or C (Fig 6). This contrasts with the relatively balanced number of genome encoded tRNA for codons ending in G or A, including tRNA for the reassigned codons UAA and UAG. Only general statements can be made about the tRNA pool from the genome survey, as both expression levels and modifications were not determined. One wobble rule states that tRNA with a G in the first anticodon position can pair with either C or U at the third position [58]. The genome survey suggests tRNAs exactly complementary to codons ending in U are simply absent, biasing the tRNA pool and requiring wobble or tRNA modification. For example, for phenylalanine the UUU codon is favored over UUC in transcripts at about a two to one ratio (Fig 6). However, the tRNA with an anticodon complementary to UUU is not present in the genome survey, while there are ten complementary to UUC. The only available tRNA anticodon would thus read GAA (third to first positions) and could either be modified through G to I deamination or pair with wobble.

A similar process could be at work for UGA encoding tryptophan where a perfectly matching tRNA was also absent. In mitochondria and *Escherichia coli*, UGG and UGA can both be translated using the same tRNA [54,59]. Another alternative is tRNA modification through deamination [60]. Conditional translation of UGA remains difficult to explain, particularly given that only subtle differences in nucleotide bias were observed when comparing the nucleotides around UGA as tryptophan and UGA as stop (Fig 4B) [61]. Short 3’ UTR length as was found in ciliates is not the only determining factor here because in general *Amoebophrya* sp. ex *K*. *veneficum* 3’ UTR are substantially longer than the examples in ciliates (Table 2). All of these clues could be seen as either cause or effect–for example mRNA containing alternative codons may be less efficiently translated due to codon bias while the ribosome pauses at apparent stop codons, allowing for some control over expression at the level of translation, or AT bias may be the primary influence on codon preference and is balanced with other mutation pressures across different genes. Comparisons between different *Amoebophrya* strains and with environmental expressed sequences [4] may help to further test concrete hypotheses. The enigmatic reputation of syndineans, with many lineages known exclusively from environmental sequencing [1,2], is enhanced by the discovery that in at least one strain of *Amoebophrya* all three typical stop codons are used to encode amino acids, and that one, UGA is contingently used as a stop codon.

## Supporting information

S1 Fig Histogram of AT % in *Amoebophrya* sp. ex *Karlodinium veneficum* transcriptome data. (PDF)

S2 Fig Nucleotide frequency around UGA as tryptophan or UGA as stop. (PDF)

S3 Fig Gene ontology terms associated with sequences containing typical stop codons. (PDF)

S4 Fig Predicted tRNA secondary structures. (PDF)

S1 Spreadsheet S1 Spreadsheet of codonw output and tRNA scan results. (XLSX)
