# Supplementary material for: A precedented nuclear genetic code with all three termination codons reassigned as sense codons in the syndinean Amoebophrya sp. ex Karlodinium veneficum
Source: PLoS One. 2019 Feb 28;14(2):e0212912. doi: 10.1371/journal.pone.0212912 (PMC6394959; doi:10.1371/journal.pone.0212912)

S1 fig. A histogram showing the number of sequences and their AT content for the *Amoebophrya* sp. ex *Karlodinium veneficum* host-parasite RNA-seq dataset over 500 bases after assembly (black line and left axis) and for those sequences with high (>90%) identity to an uninfected host dataset (red line and right axis). The assembled data for *Amoebophrya* sp. ex *Akashiwo sanguinea* assembled host-parasite data is shown with a blue line (left axis).

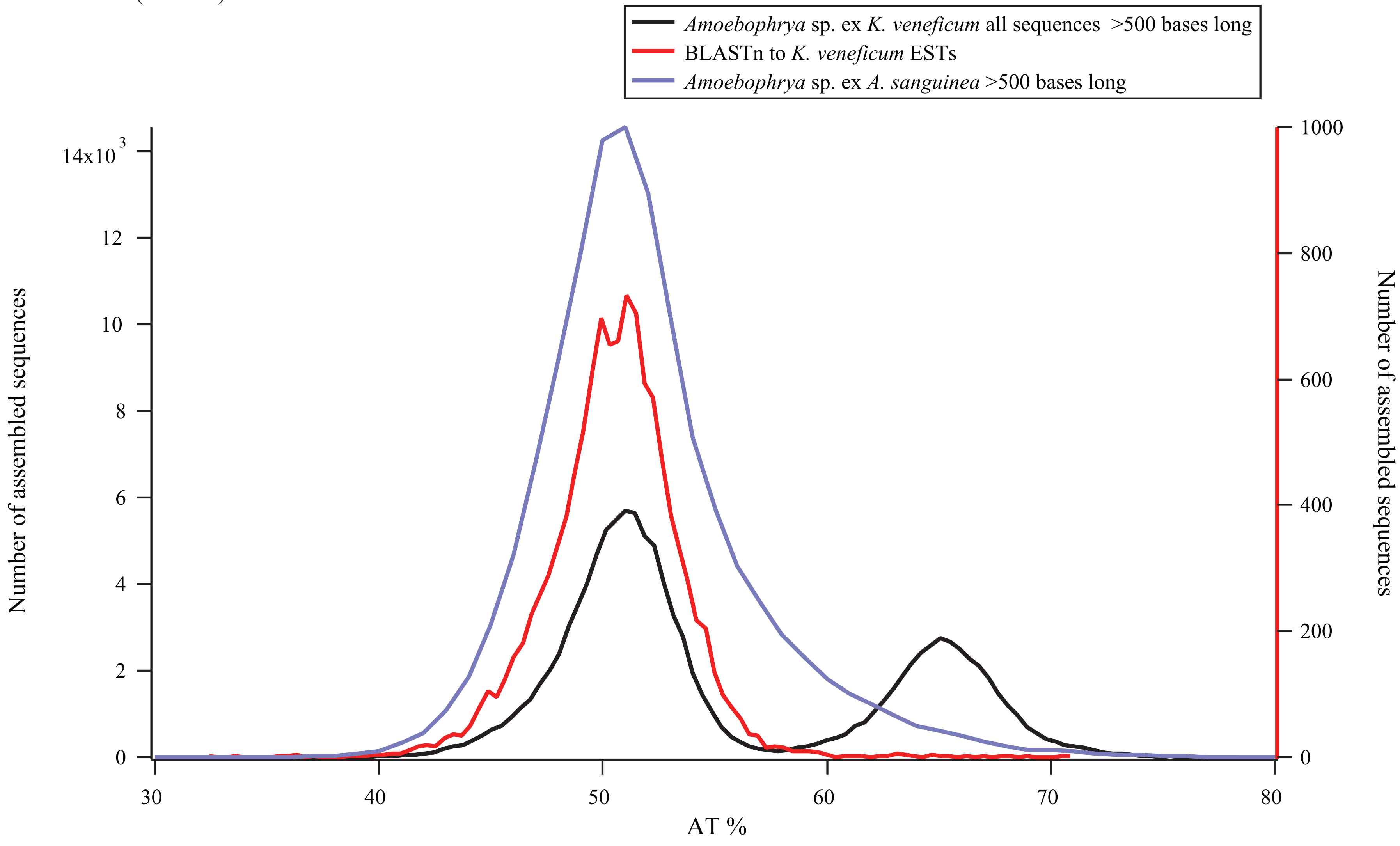

Supplement: S1 Fig — (PDF) [file pone.0212912.s001.pdf]
