# Supplementary material for: A precedented nuclear genetic code with all three termination codons reassigned as sense codons in the syndinean Amoebophrya sp. ex Karlodinium veneficum
Source: PLoS One. 2019 Feb 28;14(2):e0212912. doi: 10.1371/journal.pone.0212912 (PMC6394959; doi:10.1371/journal.pone.0212912)
Supplement: S2 Fig — (PDF) [file pone.0212912.s002.pdf]

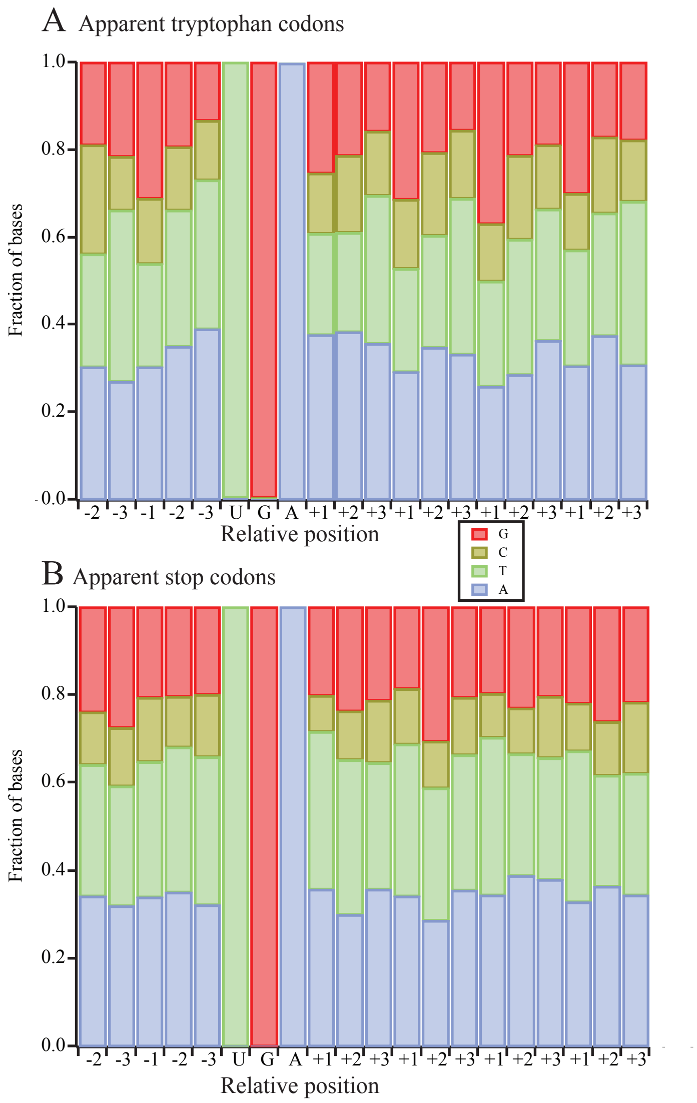

S2 fig: Nucleotide frequency around the two senses of the UGA codon. For apparent coding UGA extracted from 333 sequences, there is a consistent AT bias increase from first to third positions, which would be expected for an AT biased coding sequence. Comparing these frequencies with by codon positions within the same open reading frame showed only subtle changes in frequency. UGA-A was 37% versus a mean of 30% for first codon positions and UGA-G was less frequent 25% with a mean of 31% for first positions. For C and T there was little difference (C 14% in first positions and first position after a UGA codon; T was 26% in first positions, and 23% of first position after UGA codons). For UGA likely used as stop, a total of 457 conserved genes met the criteria described in the methods and were used to find local nucleotide context. Although C was infrequent at 14% in first and third codon positions of these 457 genes, this nucleotide was even less frequent in the first position after the stop codon (8 and 11% of bases in +1 and +2 positions). Similarly, the position after a stop was enriched for T (36 % vs. 26% for first codon positions) and had fewer G (20% versus 31%), while the A content was slightly increased (36% vs 30%). In the upstream direction, positions before UGA as tryptophan were compared with third codon positions and only showed differences of 1—2 % compared to other third positions, while for UGA as stop showed a 6% enrichment of A (39% prior to UGA versus 33% in third positions), and slight decreases in G (3% less) and A (2 % less).
