# Supplementary material for: A precedented nuclear genetic code with all three termination codons reassigned as sense codons in the syndinean Amoebophrya sp. ex Karlodinium veneficum
Source: PLoS One. 2019 Feb 28;14(2):e0212912. doi: 10.1371/journal.pone.0212912 (PMC6394959; doi:10.1371/journal.pone.0212912)
Supplement: S3 Fig — (PDF) [file pone.0212912.s003.pdf]

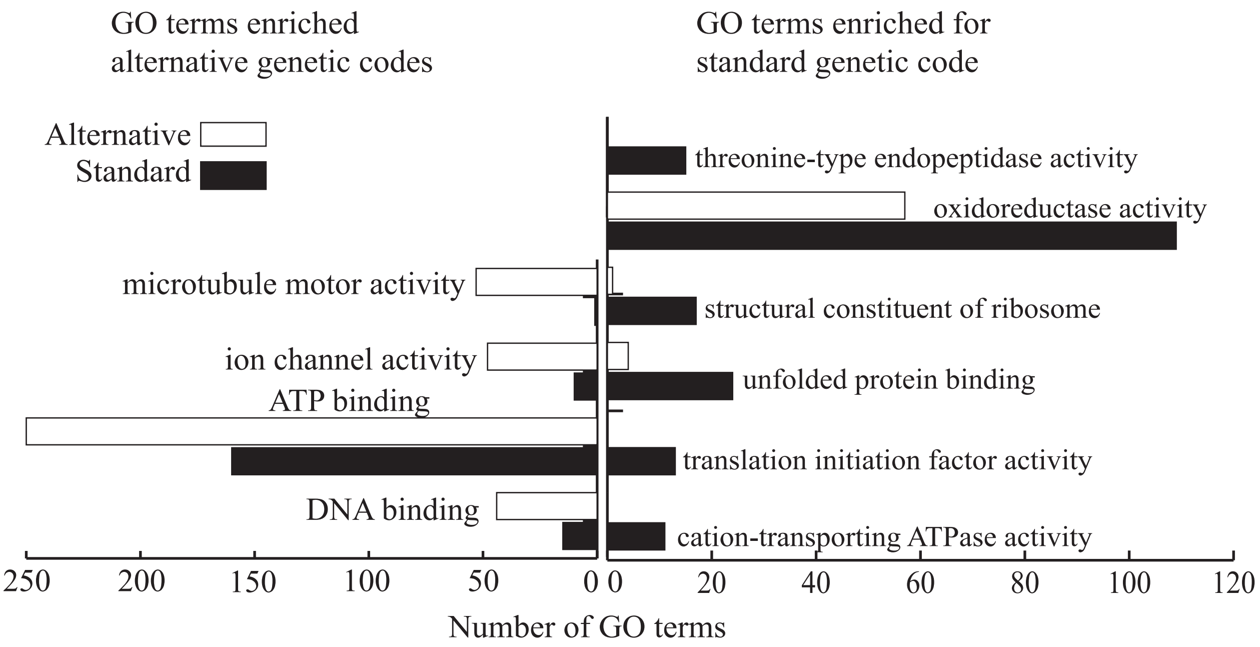

S3 fig Annotations associated with different codons. The number of gene ontology (GO) terms for different activities when comparing 1988 sequences with optimal scores using the standard genetic code to 1664 sequences with optimal scores using either NCBI genetic codes 4 or 6, or both. Only terms with significant bias in the Fisher exact test between categories are shown.
