# Supplementary material for: A precedented nuclear genetic code with all three termination codons reassigned as sense codons in the syndinean Amoebophrya sp. ex Karlodinium veneficum
Source: PLoS One. 2019 Feb 28;14(2):e0212912. doi: 10.1371/journal.pone.0212912 (PMC6394959; doi:10.1371/journal.pone.0212912)

S4 fig. Predicted secondary structures for tRNAs with anticodons complementary to typical stop codons

Contig: 313994, 42,272 bases
Position: 8971-8900
tRNAscan-SE score 53.18
Top isoform scores: histidine 65.4 , glutamine 61.2
Codon: UAG

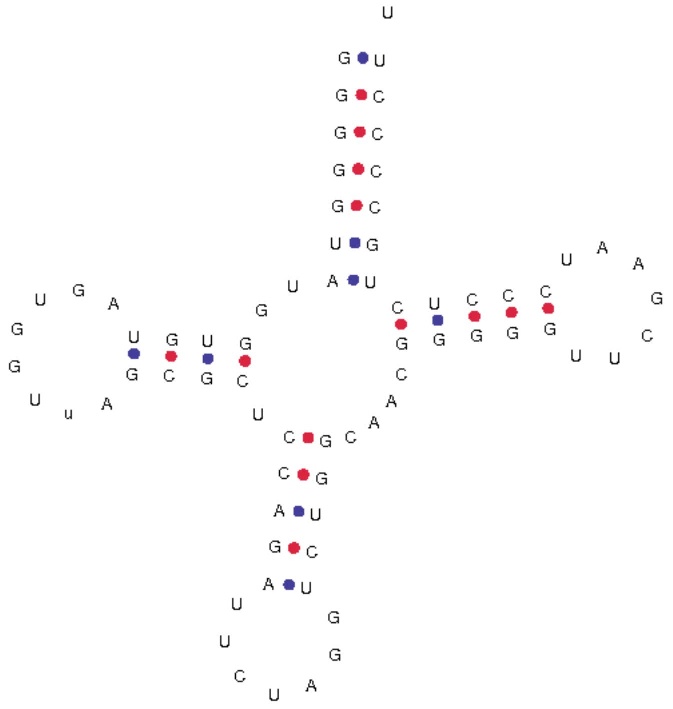

Contig: 313994, 42,272 bases
Position: 9610-9539
tRNAscan-SE score 53.59
Top isoform scores: histidine 64.7, glutamine [illegible]
Codon: TAA

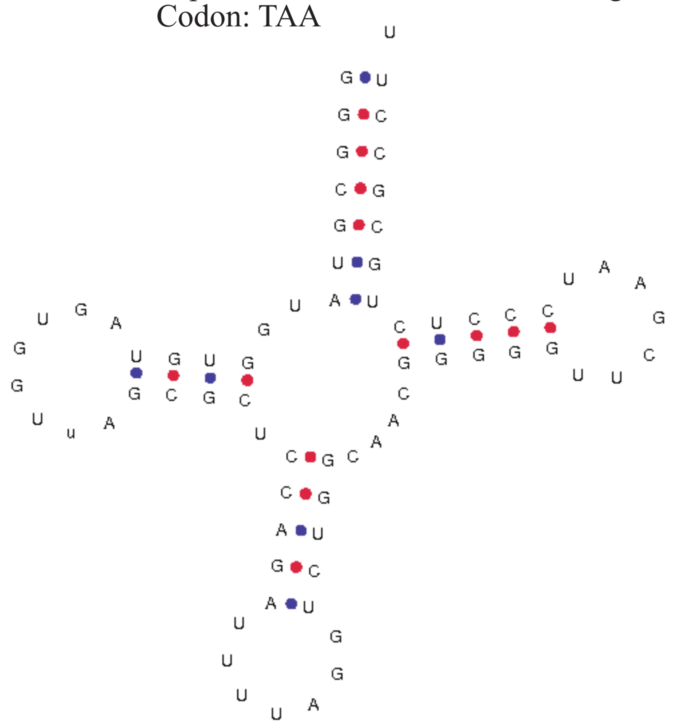

Contig: 134392, 1682 bases
Position: 412 -341
tRNAscan-SE score: 49.44
Top isoform scores: glutamine 67.0, histidine 49.4
Codon: UAG

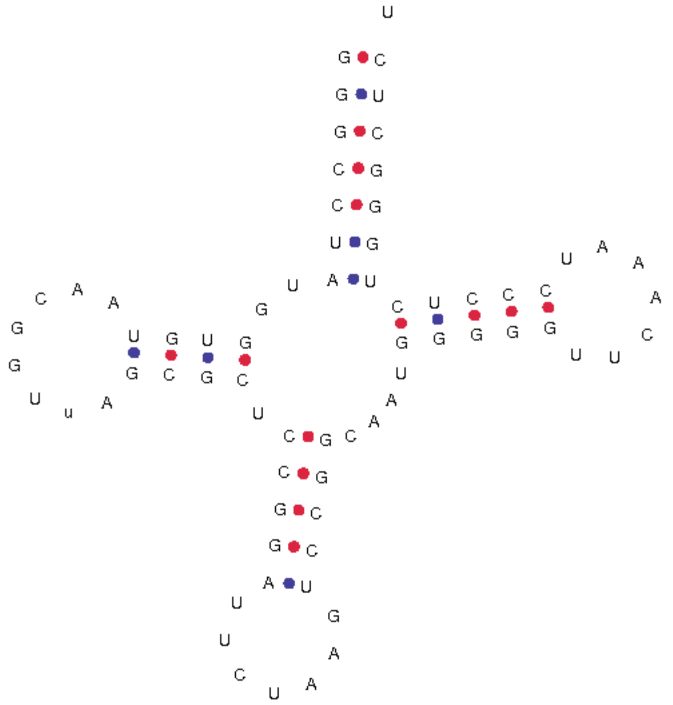

Contig: 317990, 1633 bases
Position: 1152-1224
tRNAscan-SE score: 26.54
Codon: UGA

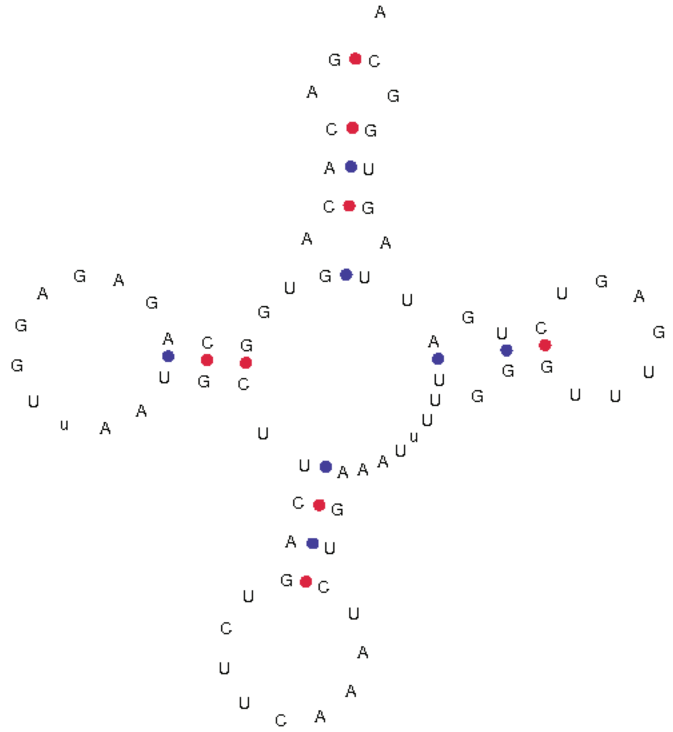

Supplement: S4 Fig — (PDF) [file pone.0212912.s004.pdf]
